# Progress and Challenges toward the Development of Vaccines against Avian Infectious Bronchitis

**DOI:** 10.1155/2015/424860

**Published:** 2015-04-14

**Authors:** Faruku Bande, Siti Suri Arshad, Mohd Hair Bejo, Hassan Moeini, Abdul Rahman Omar

**Affiliations:** ^1^Department of Veterinary Pathology and Microbiology, Faculty of Veterinary Medicine, Universiti Putra Malaysia (UPM), 43400 Serdang, Selangor, Malaysia; ^2^Department of Veterinary Services, Ministry of Animal Health and Fisheries Development, PMB 2109, Usman Faruk Secretariat, Sokoto 840221, Sokoto State, Nigeria; ^3^Laboratory of Vaccine and Immunotherapeutics, Institute of Bioscience, Universiti Putra Malaysia (UPM), 43400 Serdang, Selangor, Malaysia; ^4^Department of Virus-Associated Tumours (F100), German Cancer Research Centre, Im Neuenheimer Feld 242, 69120 Heidelberg, Germany

## Abstract

Avian infectious bronchitis (IB) is a widely distributed poultry disease that has huge economic impact on poultry industry. The continuous emergence of new IBV genotypes and lack of cross protection among different IBV genotypes have been an important challenge. Although live attenuated IB vaccines remarkably induce potent immune response, the potential risk of reversion to virulence, neutralization by the maternal antibodies, and recombination and mutation events are important concern on their usage. On the other hand, inactivated vaccines induce a weaker immune response and may require multiple dosing and/or the use of adjuvants that probably have potential safety risks and increased economic burdens. Consequently, alternative IB vaccines are widely sought. Recent advances in recombinant DNA technology have resulted in experimental IB vaccines that show promise in antibody and T-cells responses, comparable to live attenuated vaccines. Recombinant DNA vaccines have also been enhanced to target multiple serotypes and their efficacy has been improved using delivery vectors, nanoadjuvants, and *in ovo* vaccination approaches. Although most recombinant IB DNA vaccines are yet to be licensed, it is expected that these types of vaccines may hold sway as future vaccines for inducing a cross protection against multiple IBV serotypes.

## 1. Background

Avian infectious bronchitis (IB) is an economically important poultry disease affecting the respiratory, renal, and reproductive systems of chickens. Although IB was first identified in North Dakota, USA [1], epidemiological evidences confirmed the circulation of several IBV serotypes in different parts of the world. Currently, both classic and variant IBV serotypes have been identified in most countries, thus making IB control and prevention a global challenge [2, 3]. The disease is associated with huge economic losses resulting from decreased egg production, poor carcass weight, and high morbidity. Mortality rate could be high in young chickens especially with other secondary complications such as viral and bacterial infections [4].

Vaccination has been considered to be the most cost effective approach to controlling IBV infection [5]. However, this approach has been challenged by several factors including the emergence of new IBV serotypes (currently over 50 variants) that show little or no cross protection [6]. Importantly, some IBV strains to which vaccines become available might disappear as new variants emerged and thus necessitate the development of new vaccines [5]. Until recently, most IBV vaccines are based on live attenuated or killed vaccines derived from classical or variant serotypes. These vaccines are developed from strains originating from the USA such as M41, Ma5, Ark, and Conn and Netherlands, for example, H52 and H120, as well as European strains such as 793/B, CR88, and D274. However, studies have shown that vaccines against these strains often lead to poor immune response especially against local strains. Live attenuated IB vaccines have also been shown to contribute to the emergence of new pathogenic IBV variants [7, 8]. Notably, changes in geographical distribution and tissue tropism have been observed in QX-like strains that initially emerged in China and spread to cause great economic loss to poultry farmers in Asia [9], Russia [10], and Europe [11–14]. This review is aimed at describing progress and challenges associated with IBV vaccine development. Some aspects of viral-induced immune responses are discussed.

## 2. Review

### 2.1. Aetiology and Genome Characteristics

**Figure 1:**
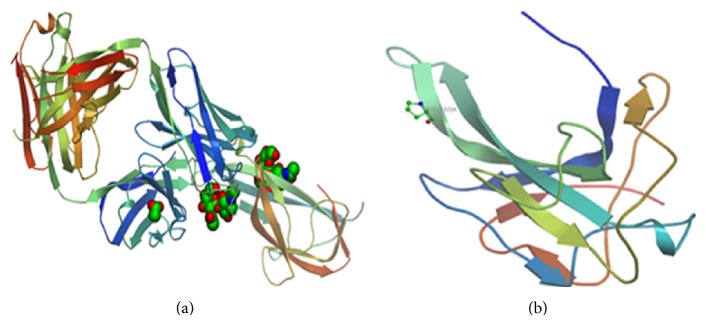
*Predicted 3-dimensional structure of S1-glycoprotein (a) and nucleocapsid protein (b), determinants of Massachusetts strain of avian infectious bronchitis virus.* Structures are drawn using SWISS homology modeller available online at http://swissmodel.expasy.org/.

Avian infectious bronchitis virus (IBV), together with Turkey coronavirus and Beluga whale coronavirus, belongs to a* Gammacoronavirus* subgroup, family Coronaviridae, order Nidovirales. Although antigenically different, members of Coronaviridae family such as SARS and MERS coronavirus share common structural protein organisation. Coronaviruses genome is made up of a single stranded enveloped RNA that measures from 27 to 32 kb, making them the largest of the RNA viruses [15]. Particularly, IBV genome has an average diameter of 80–120 nm and a typically large club of 20 nm, with heavily glycosylated spike projections. Four different genes encoding for the structural proteins are found in IBV genome. These are designated as spike (S), envelope (E), matrix (M), and nucleocapsid (N). The structural protein genes are also interspaced by genes coding for nonstructural and accessory proteins, arranged in the order of 5′ to 3′ directions as UTR-1a/1ab-S3a-3b-E-M5a-5b-N-3′-UTR-poly(A) [16]. Of the structural protein genes, the S1 and N proteins contain epitopes responsible for host immune response (Figure 1).

### 2.2. Spike Glycoprotein

The S-protein is heavily glycosylated transmembrane protein that spanned from 1,160 amino acids, giving rise to 150–200 kDa. It possessed a cleaved signal sequence, one transmembrane domain, and a short C-terminal tail [17]. IBV S-protein is made up of 3400 nucleotides posttranslationally cleaved into S1 (520 AAS residue) at the amino terminal and S2 (625 AAS residue) at the carboxyl terminal. The two glycosylated proteins (S1 and S2) are anchored in the hydrophobic region near the carboxylic part of the S2 and cleaved by furin or its related enzymes in the Golgi complex [18, 19]. Typically, S1-glycoprotein plays a role in receptor binding, while the S2 contributes aids in the fusion of the virus [20]. Of the two S-glycoprotein genes, the S1-gene is the important immunogenic component and contained epitopes responsible for neutralizing antibody [21, 22]. It also determines receptor binding as well as membrane fusion via virus-to-cell and cell-to-cell interactions [20].

### 2.3. The Nonstructural Genes' 3a, 3b, 5a, and 5b Proteins

The IBV genome possesses two small nsp genes, 3 and 5, that express three (3a, 3b, and 3c [E]) and two (5a and 5b) gene products, respectively. The 3a, 3b, 5a, and 5b proteins of IBV show a unique sequence characteristic when compared to members of group I and II coronaviruses [23]. Although the specific function of small protein remains unknown these genes are thought to contribute to virus virulence [23, 24]. Studies on the function of 5a-ns segment using reverse genetics have identified a possible link between ns-protein and virus virulence; however, their contribution to virus replication may be less relevant [25].

### 2.4. Matrix Protein

The coronavirus matrix protein (M-protein) slightly protrudes to the surface and is situated between 220 and 262 aa, which is glycosylated on the N-terminal domain [26]. Although members of group 2 coronavirus are O-glycosylated, IBV and members of group 1 coronaviruses are glycosylated with N-linked oligosaccharide molecules [27]. The role of glycosylation of M-protein is still not clear; however, using the MHV model, it was found that cell infected with MHV containing N-glycosylated M-protein induces a better interferon response compared to those infected with O-glycosylated M-protein, while nonglycosylated M-protein MHV infection resulted in a very poor interferon response [28, 29].

### 2.5. Nucleocapsid Protein

During viral replication, direct interaction occurs between N- and M-proteins [30] and similarly between N and nsp3a [31]. Similarly, an indirect interaction has been suggested between N and S as a result of S-M-protein segments interaction [30]. Nucleocapsid protein functionally binds with the genomic gRNA to form a helical ribonucleoprotein complex (RNPC), thus aiding transcription, replication, translation, and packaging of the viral genome during the replication process [32]. Coronavirus N-protein also plays role in the induction of cytotoxic T-lymphocytes response due to the presence of CTL-inducing epitopes located at its carboxylic terminus [33, 34]. In addition, novel linear B-cells epitope peptides have been mapped within the nucleocapsid N-terminal domain [35].

### 2.6. Small Envelope Protein

The IBV small envelope “E” protein is a scant protein and contains highly hydrophobic transmembrane N-terminal and cytoplasmic C-terminal domains. This protein has been suggested to be associated with viral envelope formation, assembly, budding, ion channel activity, and apoptosis [36, 37].

### 2.7. Serotypes and Strain Variations

**Figure 2:**
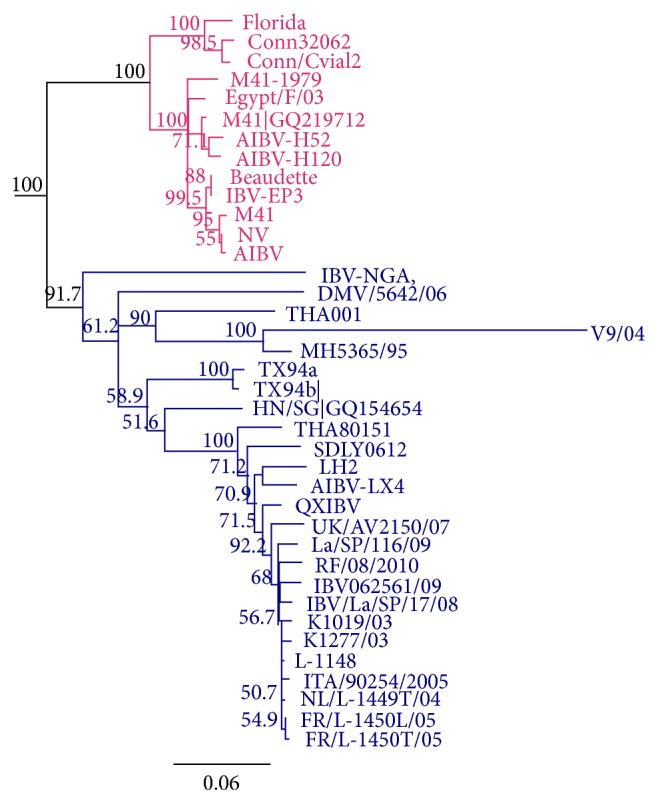
*Neighbour-joining phylogenetics showing relationship in S1-glycoprotein of classical (pink) and variant (blue) IBV strains.* The tree reliability was assessed using 1000-bootstrap confidence and branching pattern is supported by 91.7–100% bootstraps values and associated taxa show 82% pairwise identity. Phylogenetic analysis was carried out using Geneious software version R8.

Currently, there are several classical and variant IBV strains that have spread in different countries [38]. These strains may be closely or distantly related as represented in the phylogenetic tree (Figure 2). Variation may arise due to a small change as little as 5% in the S1 amino acid composition and may lead to alteration in cross protection among closely related serotypes. Thus, the nature of IBV-S1 sequences is taken into consideration in designing novel control strategies [39, 40]. Despite being first identified in USA, the classical M41 serotype and the Dutch H120 serotype are the most widely used vaccine viruses [3]. However, the World Organization for Animal Health (OIE) recommended that the distribution of IBV serotypes should influence the choice of vaccine for use in each geographic region. For example, M41, Arkansas, and Connecticut are common in USA, while 4/91 (793/B, CR88) and D274 are predominately found in Europe [41, 42]. Recently, the Chinese QX variants have emerged to cause outbreaks in Europe, Asia, the Middle East, and Africa, demonstrating a shift in geographical distribution and importance of the QX-like genotype. This variation in strain distribution is indeed a challenge to IBV control programmes. It is expected that other serotypes will continue to emerge as a result of RNA mutation and recombination that lead to viral selection pressure [3, 43]. Other local variants are common within specific regions and/or countries, but their global distribution is yet to be ascertained [44].

### 2.8. RNA Mutation and Recombination

Mutation and recombination are important phenomena that shaped coronavirus viral genomes [45]. As with most RNA viruses, mutation and recombination are two important events that alter or shape coronavirus viral genome. Consequently, a viral subpopulation may evolve as a result of these important genetic events [16, 43, 46]. Although it is difficult to ascertain how IBV genome evolved, three major theories have been hypothesised as follows: (i) the lack of RNA polymerase proofreading activity could lead to errors in RNA genome which in turn result in mutation especially in the S1 spike gene (nucleotide insertions, deletions, or point mutations). (ii) The use of vaccines especially the live attenuated vaccines type or presence of multiple infections with different IBV serotypes contributes to recombination process that favours the emergence of new IBV variants [47]. Mixture between various genetic mutants of the same coronavirus strains has been shown to generate quasispecies viruses [48, 49]. Mutations, in the hypervariable S1 domain, may affect viral subpopulation and result in new viruses with different pathogenicity as well as virulence [43, 46]. It was found that regions encoding for the nonstructural proteins 2, 3, and 16, as well as the spike glycoprotein, exhibited the highest degree of recombination [50]. Likewise, experimental passaging of IBV in the presence of other immunosuppressive viruses such as Marek's disease virus, chicken anaemia virus, and infectious bursal disease, has been suggested to affect IBV evolutionary dynamics [51].

## 3. Host Immune Response against Infectious Bronchitis Virus

### 3.1. Passive Immunity

Maternally derived antibodies (MDAs) are important components of early protection against infectious agents. It was shown that MDAs last from days to weeks, depending on the virus strain. Approximately 97% of birds with MDAs are likely to be protected against IBV infection at day one of age. However, this protection may decline to <30% by age of 7 days, thus demonstrating a limited duration of protection [52]. Adoptive transfer of antibody also has been reported to induce *αβ* associated CD8+ T-lymphocytes that protected chickens from infection with virulent IBV strain, thus signifying the role of passive immunity in IBV infection [52].

### 3.2. Innate Immune Responses

The innate immune response is important as the body's first line of defence. This response relies on pathogen-associated molecular patterns (PAMPs) through specific pattern-recognition receptors (PRRs) that are displayed on immune cells such as dendritic cells, macrophages, lymphocytes, and several nonimmune cells such as endothelial cells, mucosal cells, and fibroblasts. Importantly, the type I interferon response which is characterized by the secretions of chicken interferon alpha and interferon beta provides efficient and rapid response against viral replication through the activation of macrophages and natural killer cells which further lead to the induction of adaptive immune response. The type II interferon response which is characterized by interferon gamma secretion is predominantly produced by the activated NK cells, dendritic cells, and CD4+ CD8+ T-lymphocytes. This will further enhance leukocytes adhesion, cause NK cells activation, and increase antigen presentation on the surface of APCs (macrophages and dendritic cells) and subsequently causes the expression of MHC-I molecules and the development of adaptive response [53].

Specifically, the toll-like receptors (TLRs) such as the TLR4, TLR5, TLR15, and TLR16 are involved in the innate sensing during viral infections [54]. As with SARS virus and mouse hepatitis virus (MHV), significant upregulation of TLR4 has been observed in IBV infection, suggesting its role in coronavirus infection irrespective of the host species involved [55]. In IBV infection, innate immune response has been associated with the secretion of type I interferon in the trachea, lungs, and kidney shortly after contact with the virus [56]. This response however depends on the virulence of IBV as well as the host adaptability of the viral strain [57]. Chicken type I interferons play important roles in the inhibition of viral replication probably through interaction with TLRs molecules and pattern-recognition receptors (PPRs) that are crucial in detecting viral entry into the cells and for bridging innate and adaptive immune responses [58–60]. This is supported by the alteration in TLRs expression, especially TLR2, TLR3, and TLR7 observed in the trachea, lungs, and kidney following IBV infection [60, 61].

Administration of synthetic oligodeoxynucleotides (CpG ODNs) led to a significant increase in the expression of interferon gamma (IFN-gamma), interleukin 1-beta, IL-6, IL-8, and oligoadenylate synthetase [62]. Similarly, transcriptional analysis and cytokine profiling revealed that IL-1*β*, MIP-1*β*, and IFN signalling pathways may serve as a bridge between innate and adaptive immunities following IBV vaccination [63]. The mechanism through which IBV induces antiviral responses is very complex. However, it was suggested that involvement of the JAK-STAT pathway and upregulation of genes related to immune response such as STAT1, MYD88, IRF1, and NFKB2 are crucial to the host immune response whereas upregulation of genes related to viral protein synthesis such as elF1 helps the virus to evade immune defences [60, 64].

### 3.3. Humoral Response

Humoral immune response is associated with the inhibition of viral replication and has been shown to correlate with IBV-specific antibody titre. Antibody response following IBV vaccination has been demonstrated in serum, tracheal swabs, and lacrimal secretion [6]. Studies have shown that both systemic (IgM and IgG) and mucosal (IgA) antibodies are essential determinants for effective clearance of the circulating virus [65]. In addition, IgA, being the major immunoglobulin molecule for mucosal response, plays a role in antibody homing at tracheal or other mucosal points of viral entry [66]. Remarkably, IgM appeared 5 days after infection (dpi), peaked at days 8–10, and disappeared around 18 days after infection, while local responses correlate with increased IgG levels and subsequent clearance of the virus [56].

### 3.4. Cell-Mediated Immune Response

The N-gene specific protein response is associated with the induction of CTLs that are responsible for clearance of IBV-infected cells [34, 67]. The CTLs response peaked after 10 days, correlating with a decrease in clinical signs and viral clearance from lungs [68]. A significant increase in the CD4+ and CD8+ T-cells has been reported following vaccination with S1-gene specific IBV vaccines; thus S1-gene importantly plays role in cell-mediated immune response [69]. Viral clearance may be associated with an increase in the expression of granzymes A during primary IBV infection and subsequent activation of NK cells that aid in direct or targeted killing of IBV-infected cells [70].

### 3.5. Mucosal Immune Response

Despite advances in the understanding of mucosal immunology, much is yet to be learned about mucosal immune responses in birds. IBV replication at Harderian glands (HG), conjunctiva influences the development of the mucosal immune response which is characterized by the secretions/production of the specific IgA. This response has been further linked with the lymphoid expansion at the head associated lymphoid tissues (HALTs) and subsequent induction of CTL response [71]. Ocular vaccination of chickens with a recombinant nucleocapsid protein (rN) and recombinant S1-protein- (rS-) based IBV vaccine (via eye drop) induced significant cell-mediated immune responses without booster vaccination or adjuvants. Birds vaccinated with such vaccines were shown to be protected against infection with virulent virus strain, though the level of IgA in the mucosa was higher in positive control birds receiving only H120 live attenuated vaccine [72].

## 4. Vaccines against Infectious Bronchitis Virus

### 4.1. Live Attenuated Vaccines

Live attenuated IB vaccines are the first generation IBV vaccines used to control IBV infection in the field. These vaccines are commercially available for application via drinking water or by coarse spray at 1 day or within the first week of age. Since the duration of immunity following live attenuated vaccines is short, booster vaccination is carried out with the same or combinations of other strains, 2-3 weeks after prime vaccination [73]. Most of the commercially available live attenuated vaccines are derived from virulent strains such as Massachusetts-based M41 serotype and the Dutch H52 and H120 strains, although some strains with regional or local impact have been used in different parts of the world [74–76].

Live vaccines often are used in broilers and as boosters for breeders. However, variation may exist among countries on the type of IBV vaccine strain approved for use. This should be guided by epidemiological knowledge of the locally or regionally prevalent strains. For example, in USA, the M41, H120, Arkansas, Delaware, Florida, and JMK-derived vaccines are used frequently. In Australia, the B and C strains are used; in UK/Europe vaccine strains include M41, 4/91, and CR88. In Netherlands, vaccination using D274 and D1466 is commonly practiced [74]. For logistics and economic reasons, some commercially available live attenuated IBV vaccines have been combined with other virus vaccines such as those against Newcastle disease virus, Marek's disease virus, and infectious bursal disease virus (IBDV). However, it is not clear whether the combination may influence immune response to the combined antigen [77]. Few examples of commercially available live attenuated vaccines include Nobilis IB-Ma5 (MSD Animal Health, UK) from Mass serotype; AviPro IB H120 which is also considered as Mass serotype based vaccine from Dutch H120 strain (Lohmann Animal Health, Germany); Nobilis 4-91 (MSD Animal Health, UK); Gallivac CR88 (Merial, USA) from European strains. Live attenuated vaccine, POULVAC IB QX, has also been produced against the recently endemic QX-like IBV strains (Pfizer, France).

Some of the limitations of live attenuated viral vaccines include reversion to virulence, tissue damage, and interference by MDA. Tissue damage due to live vaccines may lead to pathological disorders or secondary bacterial infections, especially in day-old chick [78]. Evidence has shown that despite efforts to reduce viral virulence by using 52 or 120 passages to produce H52 and H120 IBV vaccines, respectively, these vaccines potentially cause considerable pathology of the trachea and may lead to a severe outbreak in the field [79, 80]. Another limitation of live attenuated IBV vaccines is potential recombination between vaccine strains and virulent field strains, leading to the emergence of new IBV serotypes [7, 75, 81]. In one study, vaccination with live attenuated H120 vaccines was shown to encourage viral spread among broiler chickens, thus potentially supporting virus transmission and persistence [82]. To reduce problems associated with vaccine reversion, researchers explore the options of using reverse genetic technology to create vaccine virus that is potentially apathogenic in the host, but capable of replication and inducing immune response. This has been shown in the case of Beaudette virus carrying the S1-gene of virulent M41 IBV strains [83, 84].

### 4.2. Inactivated or Killed Vaccines

Inactivated or killed vaccines have been used either alone or in combination with live attenuated IBV vaccines [85]. These vaccines usually are administered by injection to layers and breeders at 13 to 18 weeks of age. Since inactivated vaccines do not replicate, they are unlikely to revert and cause pathological effects. However, compared to live attenuated vaccines, killed vaccines alone induce shorter immune response characterized by antibody production but not T-cell-mediated responses [34, 86]. Therefore, inactivated vaccines in most cases require priming with live attenuated vaccines, large doses of adjuvants, and/or multiple vaccinations. This may increase the costs associated with vaccine development and marketing, thus limiting their applications [5]. Being injectable, administration of killed vaccines is either difficult or impracticable in large poultry setting. Likewise, issues of injection-site reactions may also lead to carcass rejection or reduction in value [87].

### 4.3. Recombinant Vaccines

#### 4.3.1. Viral Vector-Based Vaccines

The use of viral vectors to deliver gene(s) of interest has been studied extensively. Remarkably, the ability of adenovirus vector to persist in cells without causing pathology as well as their tropism to various dividing and nondividing cells allows sustained antigen release. It is also possible to package and express different immunogenic protein subunits in vector-based vaccines without the necessary use of a whole virulent organism [88]. Experimental recombinant vector vaccines have been developed against IBV. These vaccines were shown to induce significant increase in the immune response and protect against IB disease [69].

Although advances in viral vector vaccines seem promising in providing effective immune response and for reducing the problems associated with RNA mutation as seen in live attenuated IBV vaccines [89], this technology does have limitations that include issue of preexisting immunity or maternally derived immunity that interferes with the live vector itself and reduces the uptake of the antigen by the antigen presenting cells and consequently the transgene expression as well as specific immune response [90]. Lack of proper protein folding and glycosylation in the host system and posttranslational modifications may alter the conformation and epitope arrangement that affect the immunogenicity and efficacy of the vaccine. These factors are currently given special attention in design and selection of recombinant IBV vaccines [91]. Recent study using a recombinant adenovirus vaccine containing IBV-S1-glycoprotein reported a significant antibody response that conferred 90–100% protection, against tracheal lesions following homologous and heterologous challenge with Vic S (serotype B) or N1/62- (serotype C-) IBV strains [69].

Different protein antigens have been coexpressed with genes encoding for genetic adjuvants for an enhanced immune response. In this regard, Shi et al. [92] show that a fowl pox virus vaccine expressing IBV-S1-gene and chicken interferon-*γ* gene [rFPV-IFN*γ*S1] enhances humoral and cell-mediated immune responses that protect chickens against homologous and heterologous challenge with LX4, LHLJ04XI, and LHB IBV strains. Expression of IBV-S1-gene with chicken IL-18 in a recombinant fowl pox virus vector produced a significant increase in antibody titre, CD4+, and CD8+ responses. Similarly, expression of IL-18 with IBV-S1-gene using a fowl pox virus vector (rFPV-S1/IL18) resulted in 100% (20/20) protection, compared with only 75% (15/20) protection, rates in chickens receiving a construct containing S1 alone [93].

Oral immunization of mice with adenovirus vector was shown to circumvent neutralization of the vector by preexisting or maternally derived antibody [94]. Interestingly, adenovirus vector vaccines have been shown to be promising for use in poultry oral vaccines. Oral immunization therefore has several advantages in poultry medicine such as the ease of application and reduction in stress associated with injection handling. Although vector-based oral vaccine may lead to an adequate transgene specific antibody response, improvements are needed for optimal T-cell response. Modifications of vector-based vaccines such as dose escalation, nanoparticle coating, use of dual vectors (e.g., combination of pox and adenovirus-based vectors) and/or swapping of adenovirus hexon gene have been attempted to circumvent the effect of preexisting immunity but with some degree of success and reported toxicity in other infection models [88].

Lentivirus vectors are finding ways into veterinary vaccines, although lentivirus-based IB vaccines are uncommon [95]. Overall, only simultaneous comparative studies will assist in understanding the advantages of one vector over others.

#### 4.3.2. Subunit and Peptide-Based Vaccines

This technology requires the use of a segment or parts of the viral protein to induce specific immune response. While subunit vaccines are derived from pathogen protein or polysaccharide, peptide vaccines are made from pathogen peptides or a portion of the genome coding for immunogenic epitope [95]. Epitope within S1- and N-gene has been targeted for the induction of neutralizing antibodies as well as CTL responses, respectively [22]. For example, a study has demonstrated that synthetic epitope peptide corresponding to S20-S255 reacted well with polyclonal antibodies against various IBV strains, thus demonstrating its potential applications for broad-based IB vaccines [96]. These broad vaccines have also been mapped between 19 and 69 as well as 250 AAS sequences within the receptor binding domain whose N-terminal plays role in viral entry [97].

Although, at experimental stages of development, synthetic and peptide vaccines have been shown to be promising in the control of IBV, some researchers have focused on developing multiepitope peptide vaccines for use against wide range of IBV serotypes. Recently, Yang et al. [98] have developed an IBV vaccine based on the multiple epitopes from S1- and N-protein genes. Expression analyses and immunization study using the designed synthetic peptides yielded significant humoral and cell-mediated immune responses that resulted in >80% protection after challenge with virulent virus. In another development, a* Lactococcus lactis* bacterial system was used to deliver peptide vaccines orally and this approach was also reported to induce mucosal immune response [99, 100].

#### 4.3.3. Plasmid DNA Vaccines

Unlike recombinant vector-based vaccines involving a live vector, DNA vaccines use a plasmid containing the gene(s) that code for an immunogenic protein(s) of interest [101]. Until recently, no licensed poultry DNA vaccine is commercially available; however, this technology has gained considerable attention, and several products are at various developmental or experimental trial stages [102]. A DNA vaccine designated pDKArkS1-DP has been developed, based on the S1-genes of Arkansas IBV serotypes. Vaccination via* in ovo* route, followed by immunization with a live attenuated vaccine at 2-week intervals, resulted in a significant immune response and 100% protection against clinical disease. On the other hand, birds receiving either* in ovo* DNA vaccination alone or live attenuated vaccine alone had ≤80% protection after challenge with a virulent IBV strain [103].

Apart from* in ovo* DNA vaccinations, other novel approaches have been evaluated. For example, intramuscular injection of a liposome-encapsulated multiepitope DNA vaccine designed from S1, S2, and N regions resulted in increased numbers of CD4+, CD3+, and CD8+, CD3+ cells, and a protective immune response in 80% of the immunized birds. Some of the advantages of epitope-based vaccines include the ability to package several immunogens in a small delivery system for targeted antibody and CTL responses [104].

Enhancement of a vaccine-induced immune response was achieved by coadministration of a DNA vaccine encoding for IBV nucleocapsid or S1-glycoprotein genes with IL-2 [105] or chicken granulocyte-macrophage stimulating factors (GM-CSF), respectively [106]. In both cases, significant increase in the humoral and cell-mediated immune responses has been reported. However, S1-encoded DNA vaccines resulted in a better immune response and accorded 95% protection that was slightly higher compared to the N-gene-encoded plasmid. In another study, a multivalent IBV-DNA vaccine encoding for the S1-, N-, and M-proteins was developed [98, 107]. The efficacy and protective capacity of each gene specific IBV-DNA vaccine were shown to improve when a cationic liposome carrier was used. A similar result was obtained through boosting with an inactivated vaccine [107].

DNA vaccines have some limitations including route of administration, since most DNA vaccines are administered by injection, thus making their application difficult in large commercial poultry [108]. However, challenges related to the route of DNA vaccines administration could be overcome using* in ovo* DNA vaccination at the hatchery [103] or by giving vaccines in drinking water or as a spray vaccine. A nanoparticle-mediated DNA delivery will assist in protecting the vaccine against enzymatic degradation and enhances their availability at mucosal surfaces for mucosal response [71]. Since DNA vaccines could be used in the presence of maternal antibodies, their usage in poultry could be used to overcome challenges associated with vaccination of young chicks against IBV infection. Other advantages of DNA vaccines include the induction of both antibody and T-cells immune response, safety, ability to express multiple proteins, thermostability, and cost of production. They could be produced within a short period, thus enabling handling of the emerging virus threat. Moreover, modifications with cytokines adjuvant favour their choice in the control of infectious diseases of poultry [109].

### 4.4. Reverse Genetic Vaccines

**Figure 3:**
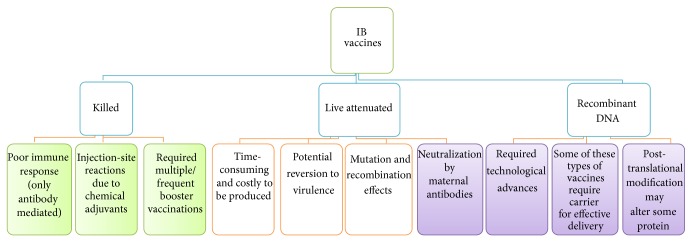
Summary of major IB vaccines and important limitations associated with the vaccine types.

A reverse genetic vaccine involved a new technology of manipulating one or more viral genes. Recently, this technology has been employed to modify IBV vaccine candidates [24, 110, 111]. For example, a recombinant, BeauR-IBV vaccine has been constructed recently by substituting the antigenic S1-glycoprotein of an apathogenic Beau-IBV strain with another S1-gene from pathogenic M41 and European 4/91 strains, respectively [112, 113]. These changes resulted in protective immune responses without making the new BeauR strain pathogenic [113, 114]. Similarly, Zhou et al. [84] have constructed a modified H120 (R-H120) virus that was found to retain some of its biological activities when rescued after 5 passages in embryonated chicken eggs. Interestingly, a vaccine using this strain has been reported to elicit a high level of haemagglutination inhibition (HI) antibody titre and a comparable protection rate compared with an intact H120-vaccinated group. The future of reverse genetic vaccines may be born out of their potentials to abrogate issues of reversion to virulence as reported with live attenuated vaccines. Development of reverse genetic IBV vaccines that may overcome neutralization in the presence of preexisting immunity, although very difficult, will surely revolutionise the use of reverse genetic-based live attenuated IBV vaccines. But whether these newer generation vaccines will increase or reduce the chances of mutation and viral selection pressure requires further studies. A summary of important limitations associated with IB vaccines is presented in Figure 3.

## 5. Expression and Delivery Systems

### 5.1. Vaccine Expression System

In recombinant or subunit vaccines, consideration is given to the presence or absence of posttranslational modification associated with the vaccine antigen. However, thorough knowledge of the chemistry and biology of the immunodominant antigen is needed to guide selection of a suitable expression system, since outcomes may differ from bacteria, yeast, mammalian, baculovirus, and plant expression systems [91]. Different expression systems have been used to generate recombinant protein antigen. An attempt, using a vaccinia virus-based IBV vaccine, failed to produce antigen enough to induce significant antibody responses in mice [115]. It was proposed that the use of vaccinia virus-based vaccines may be hindered by issues of safety regarding vaccinia virus itself, as well as its poor replication ability in avian cells [116]. In another study, a baculovirus-based vector was used to express the S1-glycoprotein of Korean nephropathogenic KM91 strain. Immunization of chickens with the KM91 vaccine resulted in 50% kidney protection following a homologous challenge [89]. Similarly, an S1-glycoprotein of IBV has been expressed in a transgenic potato under the control of a cauliflower mosaic virus (35S) promoter gene. This success could be useful in designing food-based oral IB vaccines for use in poultry [117].

An improved “BacMam” virus surface display technology, a modified strategy from baculovirus vectoring, was used recently to display the S1-glycoprotein of IBV-M41 serotype. Subsequent experimental trials with the vaccine resulted in significant humoral and cell-mediated immune responses. About 83% of the challenged birds were shown to be protected, which is comparable to 89% protection obtained in birds immunized with commercial inactivated vaccine [118].

### 5.2. Delivery System

The route of administration and delivery method used in vaccination may affect vaccine-induced immune responses, antigen presentation, and type of MHC molecule involved in the resultant response. Live attenuated IB vaccines have gained wide application via injection orally and through aeronasal spray. Killed or inactivated DNA vaccines and peptide-based vaccines are commonly used via injection routes. Some improved methods have been used to deliver recombinant proteins, plasmid DNA, and peptide vaccine. For example, an IBV-DNA vaccine carrying S1- and/or N-protein of IBV has been delivered orally using attenuated* Salmonella enterica *serovar Typhimurium strain. Interestingly, both humoral and mucosal immune responses were shown to significantly increase following oral and intranasal immunization. Vaccinated chickens were protected against homologous challenge [119]. Other approaches recorded success using a* Lactococcus lactis* bacterial system to deliver IBV vaccine, and this approach led to an efficient mucosal immune response [99, 100].

Virus-like particle (VLP) has been a new focus of interest in vaccine development. This technology utilizes the immunogenic properties of a live virus without potential to retain pathogenic effects [120]. A VLP-based IBV vaccine has been developed using the IBV-M- and IBV-S-genes. Immunization of mice with the candidate vaccines demonstrated high levels of cell-mediated immunity, comparable with the results obtained using H120 live attenuated virus vaccine. Similarly, a chimeric VLP vaccine has been synthesized using M1 protein of avian influenza H5N1 virus and fusion protein “NA/S1” derived from IBV-S1 protein and the cytoplasmic and transmembrane domains of H5N1 avian influenza NA protein. The chimeric vaccine induced significant S1-specific antibodies in mice and chickens, neutralizing antibody in chickens, and increased IL-4 secretion in immunized mice [121]. Putting together these findings, there is a huge potential for VLP-based vaccines as innovative candidate and their use may provide a delivery system for the newer IBV vaccine [120].

## 6. Conclusion

Despite spending huge amounts of money to control IB, outbreaks involving classical and newly emerging virus serotypes are constantly reported. The increasing emergence of IBV genotypes and lack of cross protective immunity have augmented the pace of interest in the development of novel IBV vaccines. Though live attenuated vaccines are still common in the field, their modification, for example, through reverse genetic technology, will be useful for reducing the effects of reversion to virulence. Viral vector vaccines have the potential to facilitate efficient protein antigen production and evoke effective immune response. However, as with live attenuated vaccines, effects of neutralization by maternal antibodies are of major concern regarding the use of vector-based vaccines, since vaccination of parent poultry breeders is practiced routinely. There is no doubt that newer generation vaccines such as the recombinant vector DNA vaccines, plasmid DNA vaccines, and multiepitope vaccines may stand as future alternatives as these vaccines have potential to deliver numerous antigens, thus producing broad-based antibody and cell-mediated immune response against numerous serotypes. Importantly, use of plasmid DNA vaccines circumvents the effect of neutralization by preexisting immunity, and their mode of action could be enhanced by delivery through different routes such as the mucosal and* in ovo* routes as well as the use of novel delivery methods such as nanoparticles and VLPs. In any case, future IBV vaccines must induce broad protection against different IBV serotypes, overcome maternal immunity, meet international safety regulations, and be easier to apply and cost effective for wider acceptance by poultry industry.

## List of Abbreviations

CTL: Cytotoxic T lymphocytes
MIP-1*β*: Macrophage inflammatory protein 1*β*
JAK/STAT: Janus kinase/signal transducers and activators of transcription
MYD88: Myeloid differentiation primary response gene 88
IRF1: Interferon regulatory factor 1
NF*κ*B2: Nuclear factor NF-kappa-B p100.
